# eIF5A is activated by virus infection or dsRNA and facilitates virus replication through modulation of interferon production

**DOI:** 10.3389/fcimb.2022.960138

**Published:** 2022-07-27

**Authors:** Rocío Seoane, Yessica Y. Llamas-González, Santiago Vidal, Ahmed El Motiam, Yanis Hichem Bouzaher, Danae Fonseca, Rosa Farrás, Adolfo García-Sastre, José González-Santamaría, Carmen Rivas

**Affiliations:** ^1^ Centro de Investigación en Medicina Molecular y Enfermedades Crónicas (CIMUS), Universidade de Santiago de Compostela, Instituto de Investigaciones Sanitarias (IDIS), Santiago de Compostela, Spain; ^2^ Grupo de Biología Celular y Molecular de Arbovirus, Instituto Conmemorativo Gorgas de Estudios de la Salud, Panamá, Panama; ^3^ Programa de Doctorado en Ciencias Biológicas, Universidad de la República, Montevideo, Uruguay; ^4^ Department of Microbiology, Icahn School of Medicine at Mount Sinai, New York, NY, United States; ^5^ Oncogenic Signalling Laboratory, Centro de Investigación Príncipe Felipe, Valencia, Spain; ^6^ Global Health and Emerging Pathogens Institute, Icahn School of Medicine at Mount Sinai, New York, NY, United States; ^7^ Division of Infectious Diseases, Department of Medicine, Icahn School of Medicine at Mount Sinai, New York, NY, United States; ^8^ The Tisch Cancer Institute, Icahn School of Medicine at Mount Sinai, New York, NY, United States; ^9^ Department of Pathology, Molecular and Cell-Based Medicine, Icahn School of Medicine at Mount Sinai, New York, NY, United States; ^10^ Cellular and Molecular Biology, Centro Nacional de Biotecnología (CNB)-CSIC, Madrid, Spain

**Keywords:** eIF5A1, hypusine, virus, dsRNA, GC7, influenza, interferon

## Abstract

Active hypusine-modified initiation elongation factor 5A is critical for cell proliferation and differentiation, embryonic development, and innate immune response of macrophages to bacterial infection. Here, we demonstrate that both virus infection and double-stranded RNA viral mimic stimulation induce the hypusination of eIF5A. Furthermore, we show that activation of eIF5A is essential for the replication of several RNA viruses including influenza A virus, vesicular stomatitis virus, chikungunya virus, mayaro virus, una virus, zika virus, and punta toro virus. Finally, our data reveal that inhibition of eIF5A hypusination using the spermidine analog GC7 or siRNA-mediated downmodulation of eIF5A1 induce upregulation of endoplasmic reticulum stress marker proteins and trigger the transcriptional induction of interferon and interferon-stimulated genes, mechanisms that may explain the broad-spectrum antiviral activity of eIF5A inhibition.

## Introduction

Polyamines have emerged as important molecules in the replication of different viruses such as Dengue virus, Chikungunya virus, Zika virus, Rift Valley fever virus, La Crosse virus, poliovirus, cytomegalovirus, coxsackievirus B3 virus, semliki forest virus or herpes simplex virus, and targeting polyamines has been suggested as a potential antiviral approach (Tuomi et al., 1980; Tyms and Williamson, 1982; Mounce et al., 2016a; Mounce et al., 2016b; Mounce et al., 2017; Mastrodomenico et al., 2019). In addition to the polyamines activities on nucleic acids and macromolecular synthesis, the polyamine spermidine is essential for the hypusination of the eukaryotic initiation factor 5A (eIF5A) (Park et al., 1981). eIF5A hypusination may be then a mechanism by which polyamines regulate virus infection. Hypusination of eIF5A is a two-step enzymatic process that post-translationally modifies eIF5A at lysine residue K50, inducing its activation (Park et al., 2010). Hypusinated eIF5A has been proposed to modulate different cellular properties including mRNA stability (Zuk, 1998; Schrader et al., 2006; Hoque et al., 2017), translation of proteins with polyproline stretches (Gutierrez et al., 2013) as well as with other non-polyproline motifs (Pelechano and Alepuz, 2017), and mRNA nucleocytoplasmic transport (Bevec et al., 1996; Kruse et al., 2000; Maier et al., 2010; Aksu et al., 2016), and it is a critical regulator of multiple biological processes including cell proliferation, embryonic development, and innate responses in macrophages upon bacterial infection (Park et al., 1997; Nishimura et al., 2005; Sievert et al., 2014; Nakanishi and Cleveland, 2016; Ganapathi et al., 2019; Gobert et al., 2020). The translation factor eIF5A and its activation have also been shown to play an important role in HIV and Filovirus replication, and its downmodulation or inhibition have been proposed as potential strategies for ebola or HIV replication control (Bevec et al., 1996; Andrus et al., 1998; Hauber et al., 2005; Hoque et al., 2009; Schroeder et al., 2014; Olsen et al., 2016). Control of HIV replication seems to be mediated by the inhibition of the nuclear export of Rev protein by eIF5A inactivation (Ruhl et al., 1993; Bevec et al., 1996; Junker et al., 1996) whereas a requirement of eIF5A for the translation of EBOV transcripts has been proposed (Olsen et al., 2018). So far, whether eIF5A plays a global role in viral infection is unknown.

In this report, we show that stimulation by double stranded RNA (dsRNA) or some RNA virus infection induce eIF5A hypusination. Activation of eIF5A is modulated by NF-κB and is essential for the efficient replication of viruses belonging to different families such as *Togaviridae, Orthomyxoviridae, Flaviviridae, Phenuiviridae*, and *Rhabdoviridae*. Finally, our data reveal that downmodulation of eIF5A1 or its inhibition trigger endoplasmic reticulum (ER) stress, as shown by increased phosphorylation of PERK and the activation of eIF2α which promotes the induction of type I interferon and type I-interferon stimulated genes, mechanisms that may contribute to the control of virus replication.

## Material and methods

### Cell lines and reagents

A549, A375, BSC40, and MDCK cells were cultured in Dulbecco´s modified Eagle´s medium supplemented with 10% fetal bovine serum (FBS), 1% L-Glutamine (G7513, Sigma-Aldrich), and 1% Penicillin-Streptomycin (P4333, Sigma-Aldrich). Vero, Vero-E6, and HeLa cells were grown in Minimal Essential medium supplemented with 10% FBS, 1% L-Glutamine, and 1% Penicillin-Streptomycin. Treatment with the spermidine analogue and competitive Deoxyhypusine Synthase (DHS) inhibitor N1-guanyl-1,7-diamine-heptane (GC7) (259545, Millipore) was carried out at a concentration of 20 µM unless otherwise indicated. IKK 16 (Abcam) and BAY 11-7082 (Sigma-Aldrich) were used at a concentration of 100 nM and 1 μM, respectively. Poly I:C (tlrl-picw, *In vivo*gen) was used at a final concentration of 5 µg/ml. Smart-pool small interfering RNAs (siRNAs) against eIF5A1 (77LQ-HUMAN_XX-0020, sieIF5A) and on-target plus non-targeting siRNAs (77D-001810-10-50, siC) were purchased from Dharmacon. A retrovirus vector expressing short hairpin RNA (shRNA) targeting p65 subunit of NF-kB was kindly provided by Scott Lowe lab (Chien et al., 2011) (Memorial Sloan Kettering Cancer Center, NY; USA).

### Transfections

Poly I:C was transfected using lipofectamine 2000 (11668019, Invitrogen) according to the manufacturer’s instructions.

### Western blotting and antibodies

Cells were collected in SDS sample loading buffer and, once separated in SDS-page gels, proteins were transferred to 0.45 μm nitrocellulose membranes. The primary antibodies used were anti-HA (#901503, Biolegend), anti-eIF5A (ab32443, Abcam), anti-phospho-eIF2α (#9721 Cell Signaling), anti-phospho-PERK (#PA5-102853, Invitrogen), anti-p65 (#3034, Cell Signaling), anti-GAPDH (sc-32233, Santa Cruz Biotechnology), anti-VSV G (a generous gift of Dr I Ventoso, CBMSO, Madrid), anti-actin (sc-4778, Santa Cruz Biotechnology), anti-influenza A virus PB2 protein (GTX125926, Gene Tex), anti-influenza A virus NS1 protein (GTX125990, Gene Tex), anti-Alphavirus E1 and NSP1 (Llamas-González et al., 2019), anti-PTV (mouse ascitic fluid kindly provided by Dr. Scott Weaver from World Reference Center for Emerging Arboviruses (WRCEVA) at University of Texas Medical Branch (UTMB, USA)), and anti-hypusine antibody (ABS1064-I, Millipore). The signal was detected using chemiluminescence and bands were densitometrically analyzed using ImageJ.

### Metabolic labelling of infected cells

Cells were infected at a multiplicity of infection (MOI) of 5, washed twice with methionine-free DMEM and labelled by incubation in methionine-free DMEM supplemented with ^35^S-methionine (100 µCi/ml) for the times indicated in the figure. Total lysates were analyzed by SDS-polyacrylamide gel electrophoresis, fixed and dried. The radioactively labeled proteins were visualized by autoradiography.

### Viral infection

Infections were carried out with vesicular stomatitis virus (VSV) of Indiana strain or recombinant VSV expressing GFP (rVSV-GFP), the mouse-adapted influenza A/PR78/34 (PR8) virus, mayaro virus (MAYV, AVR0565 strain, San Martin, Peru), una virus (UNAV, BT-1495-3, Bocas del Toro, Panama), chikungunya virus (CHIKV, Panama_256137_2014 strain, Panama), zika virus (ZIKV, 259249 strain, Panama), and punta toro virus (PTV, Adames strain, Panama). All arboviruses were a kindly gift of Dr. Scott Weaver from WRCEVA, UTMB, USA. MAYV, UNAV, CHIKV, ZIKV, PTV, and VSV were resuspended in serum-free medium, and PR8 was resuspended in 0.3% BSA in PBS. One hour after inoculation with the virus, the unbound virus was discarded and fresh medium was added. In the case of influenza virus, TPCK-trypsin (2.0 μg/ml) was added to the medium. Virus yields were measured by plaque assay in BSC40 (VSV), MDCK (PR8), Vero-E6 (MAYV and UNAV), and Vero cells (CHIKV, ZIKV, and PTV). VSV-GFP infectivity values were determined by flow cytometry (FACS) analysis using a FACSCalibur cytometer (BD Biosciences).

### Quantitative RT-PCR

Purification of total RNA was carried out using the RNeasy minikit (Qiagen), reverse transcription (RT-PCR) was performed using the reverse transcription system kit (Promega), and Q-RT-PCR was carried out using SYBR Green Power PCR Master Mix in a RealPlex 4 Thermocycler (Eppendorf). The oligonucleotides used are listed in **Table 1**.

**Table 1:** Oligonucleotides used for RT-PCR.

Oligonucleotide name	Sequence
NS1-FLU-F	5’-GGAAGGGGCAGTACTCTCGG-3’
NS1-FLU-R	5’-TTTCTGCTTGGGTATGAGCA-3’
M1-FLU-F	5’-CGCACAGAGACTGGAAGATG-3’
M1-FLU-R	5’-TGGATCCCCGTTCCCATTAA-3’
NP-FLU-F	5’-GCGTCTCAAGGCACCAAAC-3’
NP-FLU-R	5’-AACCGTCCCTCATAATCAC-3’
PB1-FLU-F	5’-ACTTACTGGTGGGATGGT-3’
PB1-FLU-R	5’-CTGAAATTGGCAACAAAC-3’
N-VSV-F	5’-CGGATGCTTCAAGAACCAGCG-3’
N-VSV-R	5’-GTCACGACCTTCTGGCACAAGAG-3’
L-VSV-F	5’-GCCAATCCCATCTCAACATCTC-3’
L-VSV-R	5’-GTATTCAATTGGTTTGTTGCCC-3’
M-VSV-F	5’-CCGTTCAGAACATACTCAGATG-3’
M-VSV-R	5’-GGTACATTGAGCATGGGAGGGG-3’
P-VSV-F	5’-CGGAGTGGAAGAGCATACTAG-3’
P-VSV-R	5’-CTCGTCGGATTCAAGCTCAGG-3’
hGAPDH-F	5’-GGAGCGAGATCCCTCCAAAAT-3’
hGAPDH-F	5’-GGCTGTTGTCATACTTCTCATGG-3’
hIFNalpha-F	5’-GCTTGGGATGAGACCCTCCTA-3’
hIFNalpha-R	5’-CCCACCCCCTGTATCACAC-3’
hIFNbeta-F	5’-ATGACCAACAAGTGTCTCCTCC-3’
hIFNbeta-R	5’-GGAATCCAAGCAAGTTGTAGCTC-3’
hISG15-F	5’-CGCAGATCACCCAGAAGATCG-3’
hISG15-R	5’-TTCGTCGCATTTGTCCACCA-3’
IFIT2-F	5’-CACATGGGCCGACTCTCAG-3’
IFIT2-R	5’-CCACACTTTAACCGRGTCCAC-3’
IFIT3-F	5’-CAGTTGTGTCCACCCTTCCT-3’
IFIT3-R	5’-CAGTTGTGTCCACCCTTCCT-3’
eIF5A1-F	5’-TGAAGATATCTGCCCGTCAA-3’
eIF5A1-R	5’-GCTGTCCTGGAGCAGTGATA-3’

### Statistical analysis

Comparison between two groups of data was carried out by Student’s t test. Values were expressed as mean +/- SF of at least three independent experiments. A p value <0.05 was considered statistically significant.

## Results

### Virus infection induces eIF5A activation

**Figure 1:**
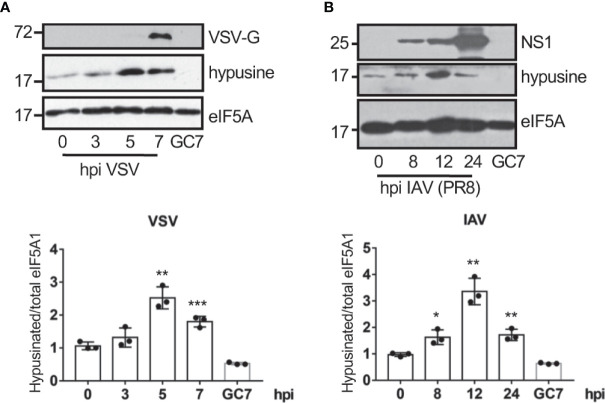
Virus infection induces eIF5A activation. Activation of eIF5A in response to VSV **(A)** or the mouse-adapted influenza A/PR78/34 (PR8) virus **(B)** infection. Hypusinated eIF5A and protein intensity bands from three biological replicates were quantified using Image J software. Hypusinated/total eIF5A ratio from each respective time were plotted. Data represent the mean and error bars of three biological replicates (lower panels). Statistical analysis was assessed by a Student’s t-test. *, P < 0.05; **, P < 0.005; ***, P < 0.0005.

We first evaluated the eIF5A status in response to virus infection. A549 cells were infected with vesicular stomatitis virus (VSV) or influenza A virus (IAV) at a MOI of 5 and at different times after infection we analyzed the activation of eIF5A using Western blot analysis with anti-hypusine antibody. We observed a significant upregulation in the levels of hypusinated eIF5A after infection with VSV (**Figure 1A**) and IAV (**Figure 1B**), indicating that virus infection induces activation of eIF5A.

### Transfection of dsRNA induces eIF5A activation

**Figure 2:**
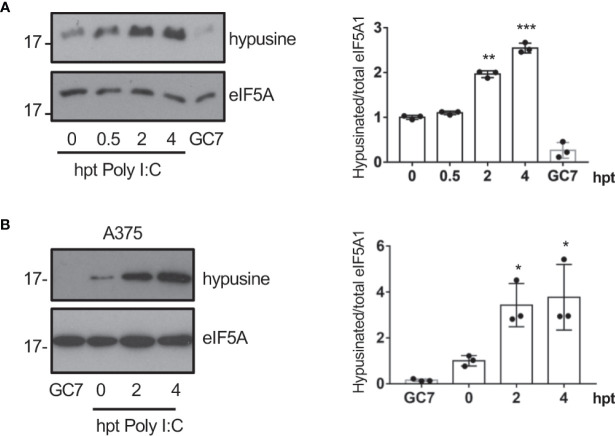
Transfection of dsRNA induces eIF5A activation. **(A)**, Activation of eIF5A in response to poly I:C transfection in A549 **(A)** or A375 **(B)** cells. Hypusinated eIF5A and protein intensity bands from three biological replicates were quantified using Image J software. Hypusinated/total eIF5A ratio from each respective time were plotted. Data represent the mean and error bars of three biological replicates (right panel). Statistical analysis was assessed by a Student’s t-test. *, P < 0.05, **, P < 0.005; ***, P < 0.0005. B, Activation of eIF5A in response to poly I:C transfection in A375 cells.

Interferon had been reported to induce depletion of polyamines (Mounce et al., 2016b) and a decrease in the hypusine synthesis (Caraglia et al., 1997) suggesting that interferon (IFN) is not involved in the virus-induced eIF5A hypusination. Most viruses produce double-stranded RNA (dsRNA) in the host cells during infection. We then assessed the activation of eIF5A at different times after cellular transfection with Poly I:C. We observed a significant increase in eIF5A hypusination after dsRNA transfection in A549 cells (**Figure 2A**), indicating that dsRNA stimulation induced eIF5A activation. Similarly, an upregulation of eIF5A hypusination was also observed in A375 cells (**Figure 2B**).

**Figure 3:**
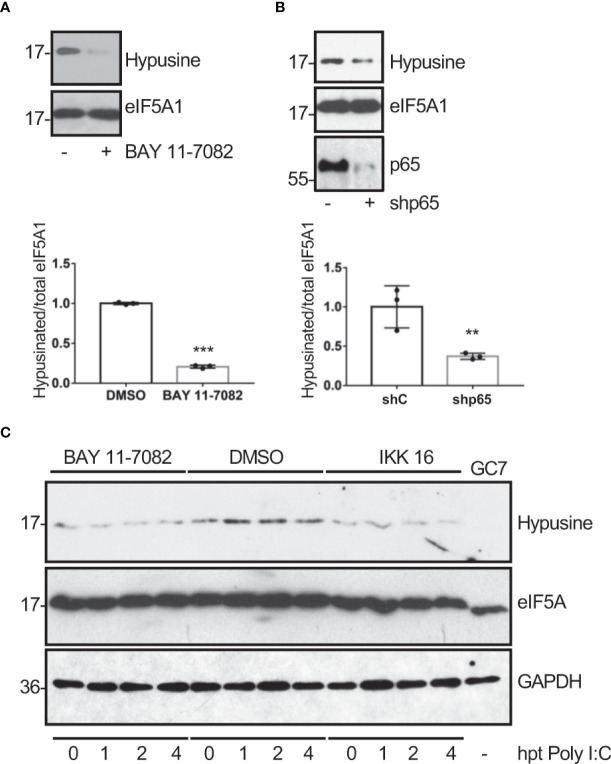
Modulation of eIF5A activation by NF-κB. Hypusine levels in A549 cells treated with BAY 11-7082 (1 μM) or DMSO for 1 h **(A)** or transduced with a retrovirus vector expressing short hairpin RNA (shRNA) targeting p65 subunit of NF-κB (shp65) **(B)**. Hypusinated eIF5A and protein intensity bands from three biological replicates were quantified using Image J software. Hypusinated/total eIF5A ratio from each respective time were plotted. Data represent the mean and error bars of three biological replicates (lower panels). Statistical analysis was assessed by a Student’s t-test. **, P< 0.005; ***, P< 0.0005. **(C)**, Activation of eIF5A in response to poly I:C transfection in A549 cells treated with BAY 11-7082 (1 μM), IKK 16 (100 nM) or DMSO, as indicated.

Ornithine decarboxylase (ODC) is a rate-limiting enzyme for polyamine synthesis that can be transactivated by NF-κB (Tacchini, 2004). Therefore, we hypothesized that the hypusination of eIF5A in response to dsRNA could be mediated by NF-κB activation. In order to test this hypothesis, we first analyzed the hypusine levels in A549 cells treated with the NF-κB inhibitor BAY 11-7082 or transfected with a shRNA plasmid to inhibit p65 expression to suppress the activation of the NF-κB pathway. Western blot analysis with anti-hypusine antibody revealed lower hypusine levels in cells treated with the NF-κB inhibitor (**Figure 3A**) or depleted of p65 (**Figure 3B**) than in control cells, indicating that the NF-κB pathway modulates hypusination in unstimulated cells. Then, we analyzed the hypusine levels in cells treated or not with the NF-κB inhibitor IKK 16 or BAY 11-7082 and then transfected with Poly I:C for the indicated times. The increase in hypusine levels observed after Poly I:C treatment in cells untreated with NF-κB inhibitors was not detected in those cells treated with IKK 16 or BAY 11-7082 (**Figure 3C**). Altogether these results suggest that NF-κB is a positive regulator of eIF5A hypusination under both basal and dsRNA-stimulated conditions.

### eIF5A activation is essential for virus replication

**Figure 4:**
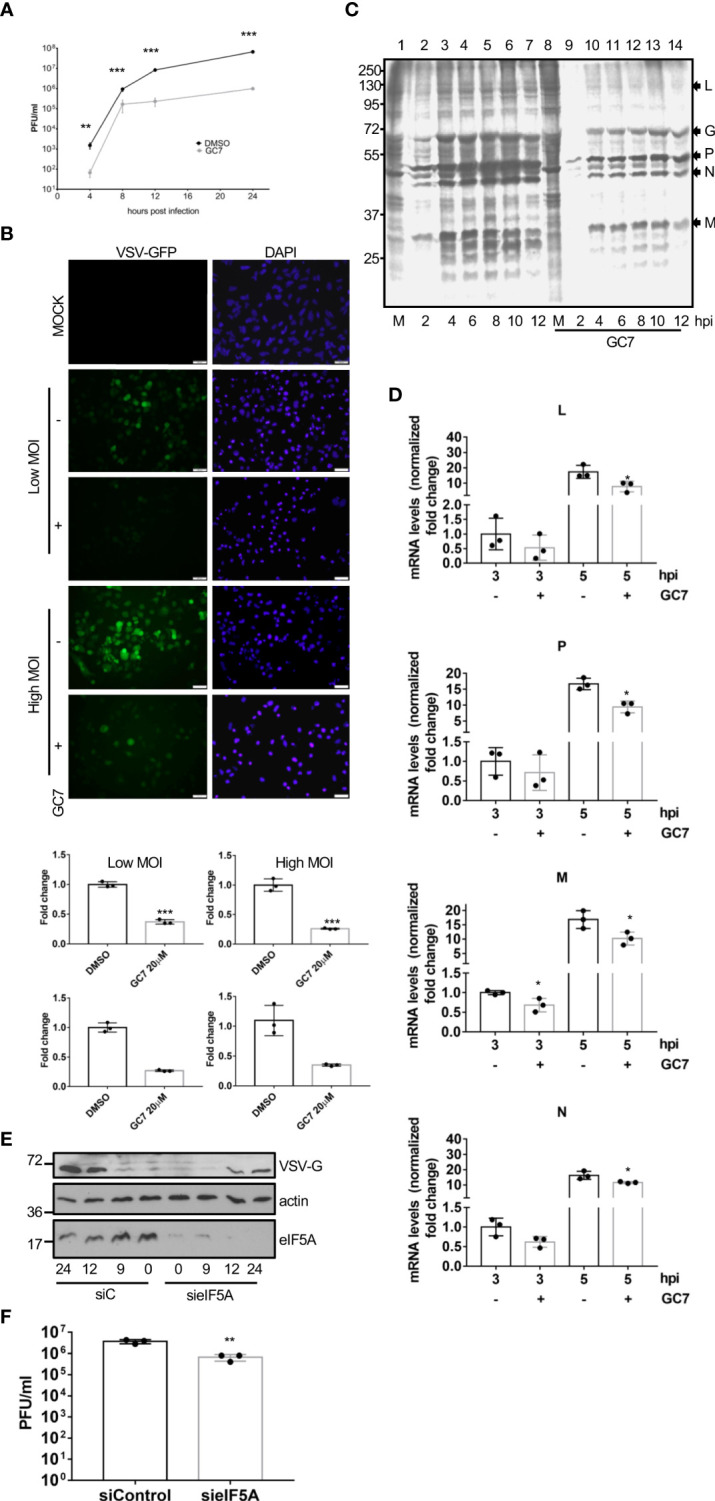
Inhibition of eIF5A impaired VSV replication. **(A)**, Effect of GC7 hypusine inhibition on VSV replication. Data represent the mean and error bars of three biological replicates. Statistical analysis was assessed by a Student’s t-test. ***, P<0.0005. **(B)**, Consequences of hypusine inhibition on VSV-GFP infection. Immunofluorescence images showing GFP and DAPI (upper panel). Quantification, by flow cytometry, of the percentage of rVSV-GFP positive cells (lower panel) in cells pretreated with GC7. Data represent the mean and error bars of three biological replicates. Statistical analysis was assessed by a Student’s t-test. *, P<0.05. **(C)**, Metabolic labelling of VSV proteins synthesized in cells treated or not with GC7. **(D)**, Consequences of hypusine inhibition on viral gene transcription at the indicated times after VSV infection. Data represent the mean and error bars of three biological replicates. Statistical analysis was assessed by a Student’s t-test. *, P<0.05. **(E)**, Effect of eIF5A1 downmodulation on viral glycoprotein G expression. **(F)**, Effect of eIF5A1 downmodulation on VSV replication. Data represent the mean and error bars of three biological replicates. Statistical analysis was assessed by a Student’s t-test. **, P<0.005.

To explore the potential role of eIF5A activation on virus replication, we first evaluated the effects of GC7 on the replication of VSV. A549 cells were treated with GC7 for 14 h and then infected with VSV at a MOI of 5. The amount of virus in the supernatant of the infected cells at 24 h after infection was determined by plaque assay. The virus titer in the GC7 treated cells was more than 2 logs less than the VSV titer in the untreated cells (**Figure 4A**), indicating the anti-VSV activity of GC7. To further evaluate its restriction on VSV replication, cells were treated with GC7 for 14 h and then infected with a recombinant VSV expressing GFP (rVSV-GFP) at a MOI of 10 or 0.1. At 24 h after infection, we evaluated the GFP signal by fluorescence microscopy as well as by flow cytometry analysis. Fluorescence microscopy revealed a decrease in the GFP signal in the GC7-treated cells (**Figure 4B**, upper panel). In addition, we detected a significant reduction in the percentage of VSV-GFP-positive cells in those cells treated with GC7 (**Figure 4B**, lower panel). To evaluate the translational shut-off as well as the kinetics of synthesis of viral proteins, metabolic labelling of infected cells with ^35^S-methionine was carried out. We observed a clear inhibition of host protein synthesis at 2 h after VSV infection (**Figure 4C**, compare lanes 1 and 2) as well as the synthesis of VSV proteins in untreated cells (**Figure 4C**). Treatment of the cells with GC7 resulted in an even stronger inhibition of host proteins synthesis at 2 h after virus infection (**Figure 3C**, compare lanes 8 and 9) and a decrease in the levels of VSV proteins synthesized in the infected cells in comparison with the untreated cells (**Figure 4C**). All together these results indicated that VSV replication requires eIF5A activation. Then, we decided to evaluate whether viral protein transcription, in addition to translation, was also affected by eIF5A inhibition. Analysis of the RNA levels of VSV proteins L, P, M and N revealed that treatment with GC7 led to a downmodulation of virus protein transcription (**Figure 4D**). Finally, to further confirm the role of eIF5A activation on VSV replication we analyzed the effect of small interference RNA (siRNA) targeting eIF5A1 on the protein synthesis and replication of VSV. Western blot analysis of A549 cells expressing or not eIF5A at different times after infection with VSV using anti-VSV-G antibody revealed a clear reduction in the viral protein synthesis in absence of eIF5A1 (**Figure 4E**). Moreover, the virus titer in the cells transfected with sieIF5A1 was significantly lower than in control cells (**Figure 4F**). All together these results indicated that the activation of eIF5A1 is critical for an efficient replication of VSV.

**Figure 5:**
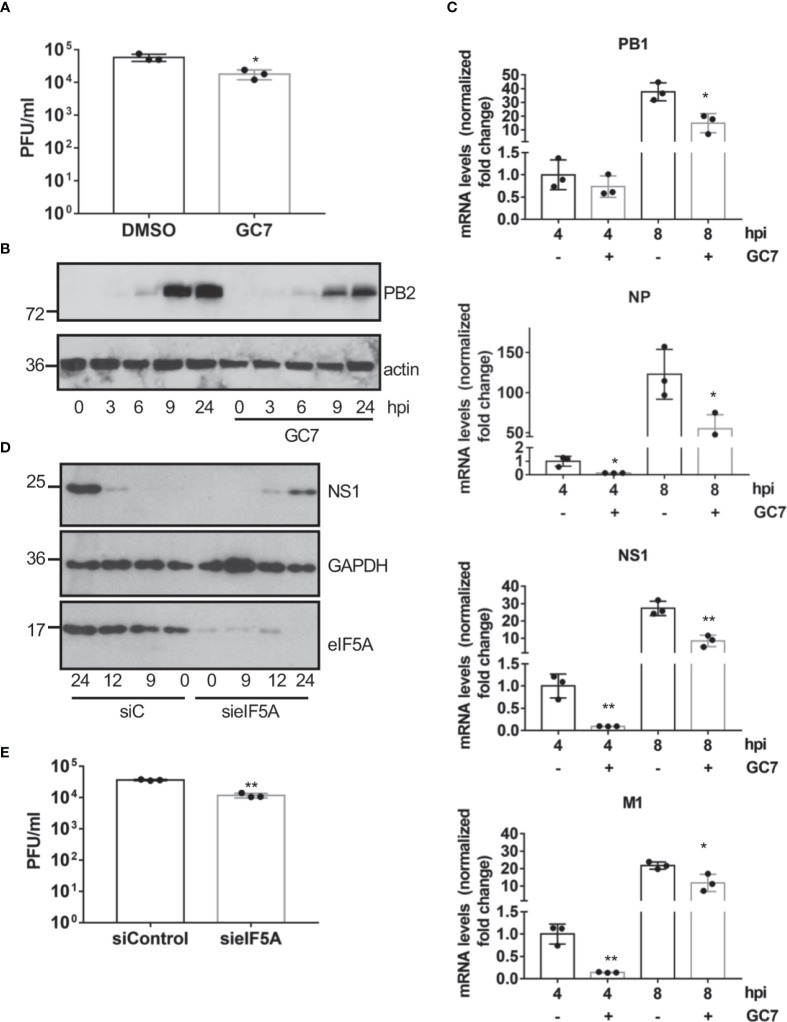
Effect of eIF5A activation on influenza virus replication. **(A)**, Effect of hypusine inhibition on IAV virus replication. Data represent the mean and error bars of three biological replicates. Statistical analysis was assessed by a Student’s t-test. *, P<0.05. **(B)**, Consequences of GC7 treatment on IAV protein synthesis. **(C)**, Consequences of hypusine inhibition on viral gene transcription at the indicated times after IAV infection. Data represent the mean and error bars of three biological replicates. Statistical analysis was assessed by a Student’s t-test. *, P<0.05; **, P<0.005. **(D)**, Effect of eIF5A1 downmodulation on NS1 IAV protein expression. **(E)**, Effect of eIF5A1 downmodulation on IAV replication. Data represent the mean and error bars of three biological replicates. Statistical analysis was assessed by a Student’s t-test. **, P<0.005.

In order to evaluate whether eIF5A activation is also required for the replication of other viruses, we decided to explore the potential antiviral activity of GC7 on IAV replication. A549 cells treated with GC7 were infected with IAV at a MOI of 5 and the virus titers at 24 hpi were then determined. We observed a significant decrease in the viral titer recovered from cells treated with GC7 relative to the one detected in untreated cells (**Figure 5A**). In addition, we also analyzed the synthesis of the viral protein PB2 in cells infected with IAV and treated or not with GC7. We observed a clear downmodulation in the synthesis of the viral protein in presence of GC7 (**Figure 5B**). Then, we analyzed the transcription of the viral proteins PB1, NP, NS1, and M1 in the presence or absence of GC7. We observed that treatment with GC7 induced a significant reduction in the transcription of the influenza A virus proteins (**Figure 5C**). Finally, we analyzed the impact of eIF5A1 downmodulation on IAV protein synthesis or replication. As shown in **Figure 5D**, downmodulation of eIF5A1 led to a reduction in the synthesis of the NS1 protein from IAV. In addition, we also observed a significant decrease in the IAV titer recovered from cells with reduced eIF5A1 in comparison with the one obtained from control cells (**Figure 5E**). All together these results indicated that eIF5A1 activation is required for efficient replication of IAV.

**Figure 6:**
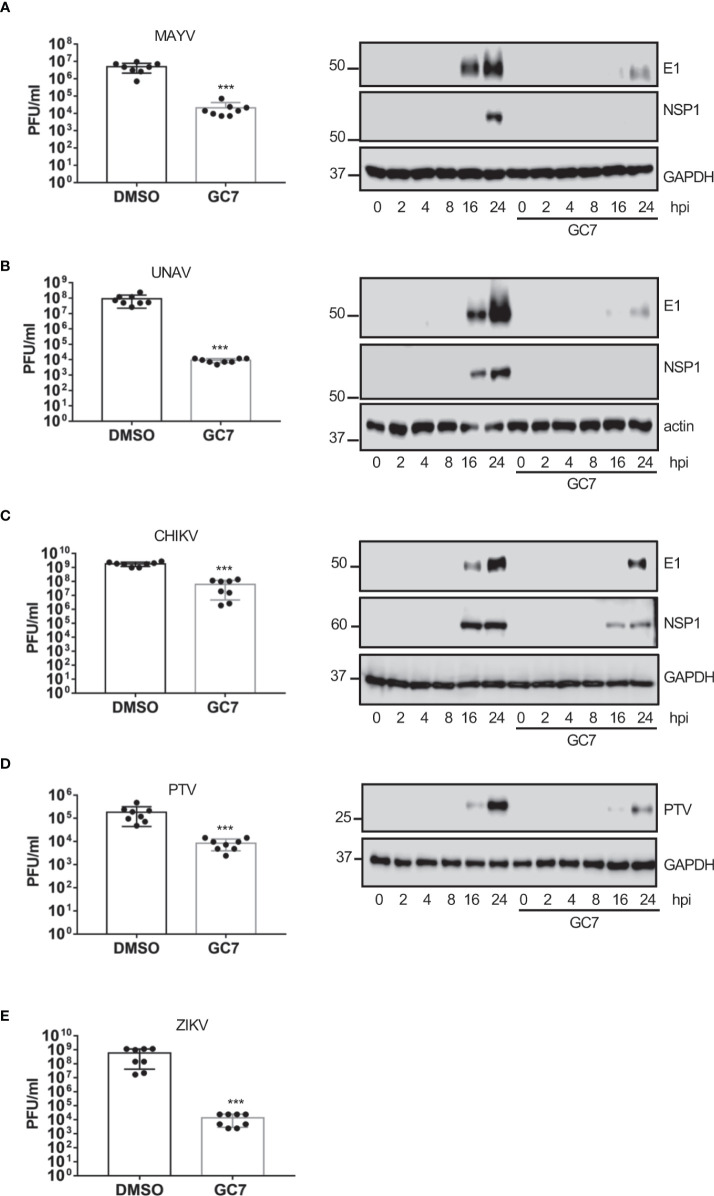
Effect of eIF5A activation on Alphavirus, Flavivirus and Phlebovirus replication. Effect of hypusine inhibition on **(A)** mayaro virus (MAYV) replication (left panel) or E1 and NSP1 viral protein synthesis (right panel), **(B)** una virus (UNAV) replication (left panel) or E1 and NSP1 viral protein synthesis (right panel), **(C)** chikungunya virus (CHIKV) replication, **(D)** punta toro virus (PTV) replication and protein synthesis, and **(E)** zika virus (ZIKV) replication. Bar graphs represent the mean and error bars of eight biological replicates (the experiment was repeated twice and in each experiment, 4 independent samples per treatment type were measured). Statistical analysis was assessed by a Student’s t-test. ***, P<0.0005.

Finally, we analyzed the effect of HeLa treatment with GC7 on the viral titer or the protein synthesis of the flavivirus zika (ZKV), Alphaviruses mayaro (MAYV), una (UNAV), and Chikungunya (CHKV), or the Phlebovirus Punta Toro (PTV). We observed a clear decrease in the synthesis of viral proteins and/or a significant decrease in the viral titer of MAYV (**Figure 6A**), UNAV (**Figure 6B**), CHIKV (**Figure 6C**), ZIKV (**Figure 6D**) and PTV (**Figure 6E**) after treatment of the cells with GC7, indicating that the activation of eIF5A plays an important role in the vital cycle of these viruses.

### Transcription of type I IFN and type I IFN-stimulated genes as mediators of the eIF5A1 antiviral activity

**Figure 7:**
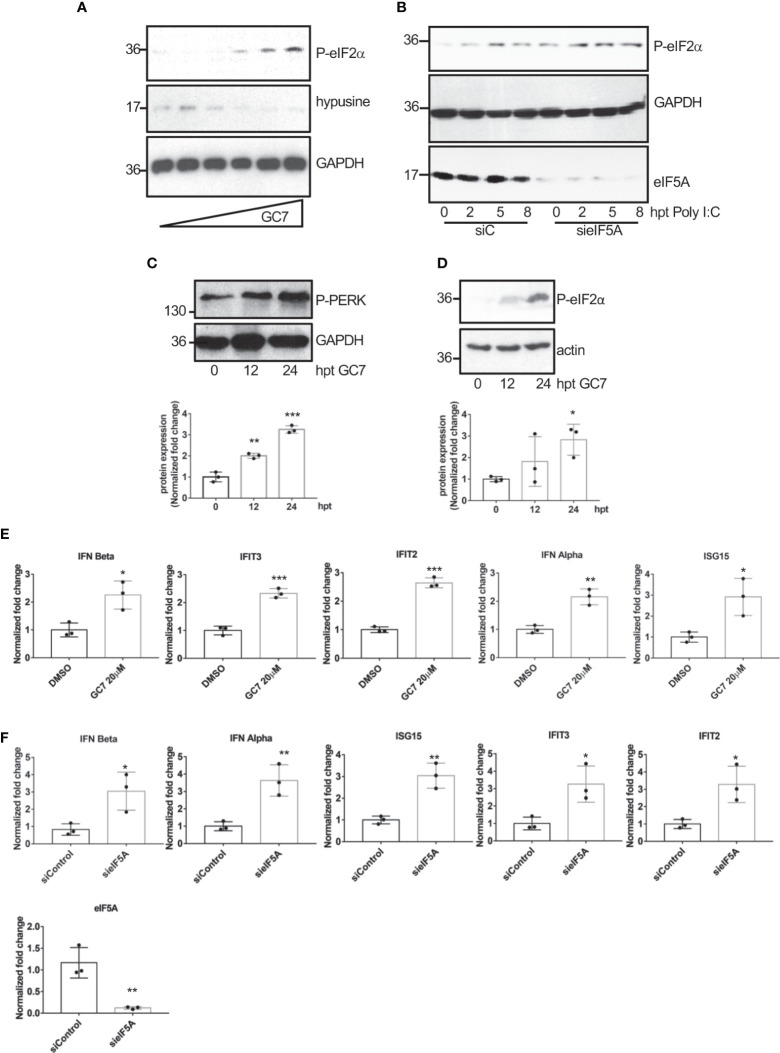
Induction of eIF2a phosphorylation and of interferon-stimulated gene expression after hypusination inhibition or eIF5A downmodulation. **(A)**, Induction of eIF2α phosphorylation after treatment with increasing dose of GC7. **(B)**, Upregulation in eIF2α phosphorylated levels after poly I:C transfection in A549 cells transfected with on-target plus non-targeting siRNAs (siC) or smart-pool small interfering RNAs (siRNAs) against eIF5A1 (sieIF5A). Induction of PERK **(C)** and eIF2α **(D)** phosphorylation at different times after GC7 treatment. Phosphorylated protein band intensity was measured and normalized to GAPDH or actin band intensity. Data represent the mean and error bars of three biological replicates (lower panels). Statistical analysis was assessed by a Student’s t-test. *, P<0.05; **, P< 0.005; ***, P< 0.0005. Expression of IFN and IFN-stimulated transcripts in A549 cells treated with GC7 for 24 h **(E)** or in A549 cells transfected with siC or sieIF5A1 (sieIF5A) for 72 h **(F)** was quantified by real-time RT-PCR and represented as fold induction over untreated cells. Data represent the mean and error bars of three biological replicates. Statistical analysis was assessed by a Student’s t-test. *, P<0.05; **, P<0.005.

The wide range of viruses affected by eIF5A1 inhibition led us to propose a general mechanism by which eIF5A1 downmodulation or inhibition of eIF5A hypusination control virus replication. It has been reported that inhibition of eIF5A hypusination in NIH3T3 cells blocks translation initiation (Landau et al., 2010) and that depletion of eIF5A leads to upregulation of endoplasmic reticulum (ER) stress marker proteins in Hela cells (Mandal et al., 2016). Therefore, we decided to evaluate the levels of ER stress markers in A549 cells treated with GC7 or depleted of eIF5A1. Western blot analysis of cells treated with different doses of GC7 revealed a significant upregulation in the phosphorylated-eIF2α protein levels in a dose-response manner (**Figure 7A**). We also observed an increase in phosphorylated eIF2α levels in cells stimulated with poly I:C after downmodulation of eIF5A (**Figure 7B**). Finally, we detected an increase in the levels of both phosphorylated PERK (**Figure 7C**) and eIF2α (**Figure 7D**) in a time-dependent manner after GC7 treatment. It is known that inhibition of protein synthesis by cycloheximide enhances and prolongs the interferon released elicited by poly I:C or endotoxin (Youngner et al., 1965; Vilček et al., 1969; Youngner, 1970; Vilček and Ng, 1971) and that ER stress induction can facilitate the transactivation and production of type I IFN (Smith et al., 2008; Martinon et al., 2010; Hu et al., 2011; Liu et al., 2012; Xue et al., 2018; Sprooten and Garg, 2020). Therefore, we decided to evaluate the transcription of interferon and interferon-stimulated genes in cells treated with GC7 or with downmodulated eIF5A1. Treatment with GC7 or eIF5A1 downmodulation induced a significant transactivation of interferon and interferon-stimulated genes (**Figures 7E, F**).

## Discussion

Here we show that VSV and IAV infection induce eIF5A hypusination. Interferon had been reported to induce depletion of polyamines (Mounce et al., 2016b) and a decrease in the hypusine synthesis required for the activation of eIF5A (Caraglia et al., 1997), suggesting that activation of eIF5A in response to virus infection was not mediated by interferon signaling. However, transfection of cells with poly I:C also induces eIF5A hypusination, indicating that the activation of eIF5A can be mediated by dsRNA produced during virus infection.

Recently, it has been found that exposition of macrophages to bacterial pathogens or to IL-4 stimulation triggers the activation of eIF5A (Puleston et al., 2019; Gobert et al., 2020). So far, this is the first demonstration that poly I:C or virus infection triggers eIF5A hypusination. It is known that stimulation of cells by dsRNA or virus infection induces NF-κB activation. NF-κB then regulates the transcription of multiple genes including ODC (Tacchini, 2004; Berrak et al., 2016), a rate-limiting enzyme for polyamine biosynthesis, suggesting that eIF5A activation triggered by dsRNA transfection or virus replication may be mediated by the NF-κB pathway. The decrease in hypusine levels in both unstimulated and dsRNA stimulated cells with inhibited NF-κB reinforces this hypothesis.

It has previously been shown that eIF5A plays an important role in the replication of HIV (Ruhl et al., 1993; Bevec et al., 1996; Andrus et al., 1998) as well as of ebola virus (EBOV) and marburg virus (Olsen et al., 2016), and in the propagation of KSHV (Fiches et al., 2022). Inhibition of hypusination has been proposed to suppress HIV gene expression at the level of transcription initiation (Hoque et al., 2009) and blocking hypusination has been suggested to limit the effective translation of mRNA transcribed by the EBOV polymerase (Olsen et al., 2018). Here we show that eIF5A activation is essential for the proper replication of viruses belonging to *Rhabdoviridae, Orthomyxoviridae, Phenuiviridae, Flaviviridae* and *Togaviridae* family, increasing the list of viruses able to exploit eIF5A for their replication, and supporting the proposal that targeting of eIF5A can be a strategy for the control of virus replication (Olsen and Connor, 2017). The broad range of viruses that relies on eIF5A for proper replication suggests that a general function of eIF5A may be at work for these viruses.

eIF5A is a translation factor required for protein synthesis and essential for the survival of eukaryotic cells. Although it has been proposed that eIF5A is required for the synthesis of a specific subset of mRNAs (Kang and Hershey, 1994; Hanauske-Abel et al., 1995; Gutierrez et al., 2013), inhibition of eIF5A1 hypusination in NIH3T3 cells blocks translation initiation through phosphorylation of eIF2α (Landau et al., 2010). Depletion of eIF5A1 also leads to global translation elongation and termination defects (Schuller et al., 2017) and to upregulation of ER stress marker proteins in Hela cells (Mandal et al., 2016). Interestingly, a recent report shows that eIF5A2 downmodulation does not regulate global protein synthesis but it controls the expression of antiviral genes and thus depletion of eIF5A2 increases the susceptibility of cells to viral infection (Farache et al., 2022). Here we show that inhibition of hypusination by GC7 treatment or downmodulation of eIF5A1 induce ER stress in A549 cells. It is known that ER stress can facilitate the transactivation and production of type I IFN (Smith et al., 2008; Martinon et al., 2010; Hu et al., 2011; Liu et al., 2012; Xue et al., 2018; Sprooten and Garg, 2020) and that inhibition of protein synthesis by cycloheximide enhances and prolongs the interferon released elicited by poly I:C or endotoxin (Youngner et al., 1965; Vilček et al., 1969; Youngner, 1970; Vilček and Ng, 1971). Therefore, we propose that treatment of cells with hypusine inhibitor or eIF5A1 silencing induces ER stress that results in the upregulation of IFN and IFN-stimulated genes, likely contributing to the control of virus replication.

## Data availability statement

The raw data supporting the conclusions of this article will be made available by the authors, without undue reservation.

## Author contributions

RS, YL-G, SV, AM, YB, and DF designed and performed the experiments. RS and CR conceived the idea. CR wrote the manuscript with input from all the authors and supervised the project. RF, AG-S and JG-S discussed and interpreted the results. All authors provided critical feedback.

## Funding

Funding at the laboratory of CR is provided by Ministry of Science, Innovation and Universities and FEDER (BFU-2017-88880-P), (PID2021-126510NB-I00), and Xunta de Galicia (ED431G 2019/02). RS and SV are predoctoral fellows funded by Xunta de Galicia-Consellería de Cultura, Educación y Ordenación Universitaria (ED481A-2020/160 and ED481A-2018/110, respectively). JG-S was supported by grants from Secretaría Nacional de Ciencia, Tecnología e Innovación de Panamá (SENACYT), grant number FID18-100, Ministerio de Economía y Finanzas, grant number 19911.012 and Sistema Nacional de Investigación (SNI from SENACYT), grant number 23-2021. This work was also partly supported by CRIPT (Center for Research on Influenza Pathogenesis and Transmission), an NIAID funded Center of Excellence for Influenza Research and Response (CEIRR, contract # 75N93021C00014) to AG-S.

## Acknowledgments

We thank Richard Cadagan for excellent technical support.

## Conflict of interest

The AG-S laboratory has received research support from Pfizer, Senhwa Biosciences, Kenall Manufacturing, Avimex, Johnson & Johnson, Dynavax, 7Hills Pharma, Pharmamar, ImmunityBio, Accurius, Nanocomposix, Hexamer, N-fold LLC, Model Medicines, Atea Pharma and Merck, outside of the reported work. AG-S has consulting agreements for the following companies involving cash and/or stock: Vivaldi Biosciences, Contrafect, 7Hills Pharma, Avimex, Vaxalto, Pagoda, Accurius, Esperovax, Farmak, Applied Biological Laboratories, Pharmamar, Paratus, CureLab Oncology, CureLab Veterinary and Pfizer, outside of the reported work. AG-S is inventor on patents and patent applications on the use of antivirals and vaccines for the treatment and prevention of virus infections and cancer, owned by the Icahn School of Medicine at Mount Sinai, New York, outside of the reported work.

The remaining authors declare that the research was conducted in the absence of any commercial or financial relationships that could be construed as a potential conflict of interest.

## Publisher’s note

All claims expressed in this article are solely those of the authors and do not necessarily represent those of their affiliated organizations, or those of the publisher, the editors and the reviewers. Any product that may be evaluated in this article, or claim that may be made by its manufacturer, is not guaranteed or endorsed by the publisher.
